# Selection and Characterization of Rupintrivir-Resistant Norwalk Virus Replicon Cells *In Vitro*

**DOI:** 10.1128/AAC.00201-18

**Published:** 2018-04-26

**Authors:** Mitsutaka Kitano, Myra Hosmillo, Edward Emmott, Jia Lu, Ian Goodfellow

**Affiliations:** a Division of Virology, Department of Pathology, University of Cambridge, Addenbrooke's Hospital, Cambridge, United Kingdom

**Keywords:** antiviral agents, drug resistance mechanisms, noroviruses, protease inhibitors, proteases

## Abstract

Human norovirus (HuNoV) is a major cause of nonbacterial gastroenteritis worldwide, yet despite its impact on society, vaccines and antivirals are currently lacking. A HuNoV replicon system has been widely applied to the evaluation of antiviral compounds and has thus accelerated the process of drug discovery against HuNoV infection. Rupintrivir, an irreversible inhibitor of the human rhinovirus 3C protease, has been reported to inhibit the replication of the Norwalk virus replicon via the inhibition of the norovirus protease. Here we report, for the first time, the generation of rupintrivir-resistant human Norwalk virus replicon cells *in vitro*. Sequence analysis revealed that these replicon cells contained amino acid substitutions of alanine 105 to valine (A105V) and isoleucine 109 to valine (I109V) in the viral protease NS6. The application of a cell-based fluorescence resonance energy transfer (FRET) assay for protease activity demonstrated that these substitutions were involved in the enhanced resistance to rupintrivir. Furthermore, we validated the effect of these mutations using reverse genetics in murine norovirus (MNV), demonstrating that a recombinant MNV strain with a single I109V substitution in the protease also showed reduced susceptibility to rupintrivir. In summary, using a combination of different approaches, we have demonstrated that, under the correct conditions, mutations in the norovirus protease that lead to the generation of resistant mutants can rapidly occur.

## INTRODUCTION

Human norovirus (HuNoV), a member of the Caliciviridae family, represents a major cause of acute viral gastroenteritis worldwide in both children and adults (1, 2). HuNoV infection can lead to severe dehydration and diarrhea, especially in the elderly or immunocompromised patients (3). The considerable economic burden incurred as a result of norovirus infections, which includes productivity losses and the burden on health care systems, highlights that HuNoV infection is a global economic problem (4). Although there is a significant unmet medical need for the prevention and treatment of HuNoV infection, no licensed vaccines or antivirals for HuNoV infection are currently available. The lack of a robust cellular system for the analysis of viral replication has hampered antiviral research against HuNoV infection for many years (5). This situation has been changed by the recent development of enterocytes derived from stem cell and B cell culture systems (6, 7). Proof of concept that these cell culture systems enable the evaluation of the antiviral effects of compounds against HuNoV has recently been demonstrated (8), but neither of these systems is suitable for large-scale screening of therapeutic compounds. In contrast, while replicon cells lack many features of an authentic viral life cycle because the viral capsid protein has been replaced with a drug resistance marker, they have been widely used for the evaluation of the activities of antivirals against HuNoV (9–11).

HuNoV belong to three genogroups, genogroup I (GI), GII, and GIV, which are further subdivided into numerous genotypes, and GII genotype 4 (GII.4) norovirus strains have been circulating worldwide since 2012 (12, 13). Murine norovirus (MNV) is included in GV of norovirus (14). The HuNoV genome is composed of three open reading frames (ORFs). ORF1 encodes the nonstructural polyprotein, which is cleaved into at least six proteins and several stable intermediates by the viral protease NS6 (15). ORF2 and ORF3 encode the major capsid protein VP1 and the minor capsid protein VP2, respectively. HuNoV, similar to other RNA viruses, has a high mutation rate that allows rapid viral evolution due to the error-prone nature of the viral RNA-dependent RNA polymerase (16, 17). This potentially permits the emergence of drug-resistant viruses during the course of treatment. In order to overcome this, the development of antiviral drugs for HuNoV infection requires both potent antiviral activity and a high genetic barrier to the generation of drug-resistant viruses, especially during treatment for persistent infection in immunocompromised patients. Information on drug resistance would facilitate drug design and would be useful for predicting and suppressing the appearance of drug-resistant viruses. To date, however, the efficiency with which resistance occurs and the mechanisms by which resistance to inhibitors might arise have yet to be described for any inhibitors against HuNoV. Therefore, we sought to examine whether resistant replicons could be identified following prolonged culture in the presence of a suitable antiviral. To achieve this aim, we utilized rupintrivir (AG7088), an irreversible inhibitor of the human rhinovirus (HRV) 3C protease. Rupintrivir has been reported to inhibit the replication of the Norwalk virus replicon in the hepatocellular carcinoma cell line Huh-7, but whether resistance can be generated remained to be determined (10). In the present study, we isolated replicon cells with reduced susceptibility to rupintrivir after several passages in the presence of rupintrivir and identified two amino acid substitutions of alanine 105 to valine (A105V) and isoleucine 109 to valine (I109V) in the viral protease. Moreover, we demonstrated that these substitutions are involved in susceptibility to rupintrivir using a previously described cell-based fluorescence resonance energy transfer (FRET) assay (18). Finally, we determined that recombinant MNV with a single I109V substitution in the protease showed reduced susceptibility to rupintrivir in cell culture. We concluded that mutations around the norovirus protease active site lead to the generation of rupintrivir resistance; however, some of these mutations appear to compromise viral fitness, at least in the context of the MNV infection model.

## RESULTS

### Selection of norovirus replicon cells with reduced susceptibilities to rupintrivir *in vitro*.

**FIG 1:**
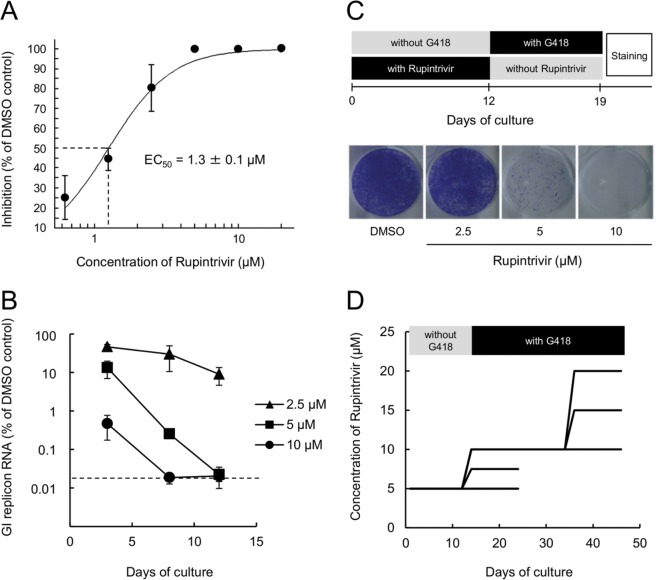
Isolation of rupintrivir-resistant HGT-NV replicon cells. (A) Dose-response curve of effect of rupintrivir on HuNoV replicon RNA levels in HGT-NV cells. The level of replicon RNA in HTG-NV cells treated with DMSO or rupintrivir was measured by quantitative RT-PCR. Inhibition was plotted as a percentage relative to that in DMSO-treated cells. Error bars represent means ± standard deviations from three independent experiments. (B) Reduction in the GI replicon RNA level over time in HGT-NV cells treated with rupintrivir in the absence of G418. The levels of HuNoV replicon RNA relative to those observed in control DMSO-treated cells were plotted. Error bars represent means ± standard deviations from three biological replicates. Dashed line, detection limit for replicon RNA. (C) Colony formation assays with HGT-NV cells treated with DMSO or rupintrivir for 12 days. Colonies were stained and photographed on day 7 after treatment with G418 (1.5 mg/ml) in the absence of rupintrivir. (D) Schematic overview of the procedure used for the repetitive cultivation of HGT-NV cells in the presence of increasing concentrations of rupintrivir. HGT-NV cells were maintained as subconfluent cultures in the presence of DMSO or rupintrivir and G418 and passaged every 2 to 3 days for 45 days.

To confirm the previously reported inhibitory effects of rupintrivir on human norovirus replication (10), we generated a human gastric adenocarcinoma cell line, HGT-NV, which stably maintained a Norwalk virus replicon encoding the neomycin resistance gene in place of the major capsid protein. The effects of rupintrivir on norovirus RNA replication in the replicon cells were evaluated by quantitative reverse transcription-PCR (qRT-PCR). Rupintrivir reduced the levels of replicon in a dose-dependent manner with a 50% effective concentration (EC_50_) value of 1.3 ± 0.1 μM and showed low cytotoxicity (50% cytotoxic concentration [CC_50_], >100 μM), which gave a calculated selectivity index (CC_50_/EC_50_) of >77 (Fig. 1A). To determine the concentration of rupintrivir sufficient to completely eliminate the norovirus replicon from HGT-NV cells, we treated the replicon cells with 2.5, 5, and 10 μM rupintrivir in the absence of G418 for up to 12 days. The replicon cells were passaged on days 3, 8, and 12, and the levels of replicon RNA were quantified by qRT-PCR. A rapid reduction of replicon RNA levels was observed in the cells treated with 10 μM rupintrivir (Fig. 1B). In addition, the levels of replicon from cells treated with either 5 or 10 μM rupintrivir were decreased to below the limit of quantification by 12 days posttreatment. To further characterize the inhibitory effects of rupintrivir, we performed a colony formation assay with replicon cells treated with rupintrivir for 12 days. After 1 week of culture without rupintrivir but with G418 (1.5 mg/ml), the colonies were stained (Fig. 1C). Rupintrivir treatment reduced the number of colonies in a dose-dependent manner. Treatment with 10 μM rupintrivir was sufficient to completely prevent colony formation, while some colonies remained when cells were treated with 2.5 or 5 μM rupintrivir.

Given the potent effects of rupintrivir on HGT-NV cells, we sought to determine whether rupintrivir-resistant replicons could be generated. To this end, HGT-NV cells were treated with 5 μM rupintrivir for 12 days in the absence of G418 selection, followed by subsequent passages in the presence of G418 with increasing concentrations of rupintrivir for up to 45 days (Fig. 1D). This approach yielded three lineages of replicon cells which were resistant to 10, 15, and 20 μM rupintrivir. To identify the mutations conferring the resistance to rupintrivir, we isolated cell clones by limiting dilution using replicon cells surviving after 45 days of culture and then evaluated the rupintrivir sensitivity of the replicon cells. In comparison to replicon cells that had been passaged in parallel in the presence of dimethyl sulfoxide (DMSO) alone and that had an EC_50_ of 1.3 ± 0.2 μM, rupintrivir-resistant replicon cells showed approximately 4- to 11-fold reduced sensitivity to rupintrivir, with EC_50_s of 5.9 ± 1.3 to 14.3 ± 1.7 μM (Table 1). Replicon cells treated with 20 μM rupintrivir exhibited the highest levels of resistance compared with those treated with 10 or 15 μM rupintrivir. Sequence analyses of the NS6 protease region of the replicon from the resistant clones revealed two amino acid substitutions: alanine 105 to valine (A105V) and isoleucine 109 to valine (I109V). All clones treated with 10 μM rupintrivir had the I109V single amino acid substitution, while the A105V/I109V double amino acid substitution was observed only in clones treated with 15 and 20 μM rupintrivir. The A105V single amino acid substitution alone was not observed in any of the clones sequenced.

**TABLE 1:** Susceptibility of HGT-NV cells to rupintrivir after repetitive cultivation

Rupintrivir concn (μM) at final passage or control treatment	Clone no.	Mean EC_50_ ± SD^*a*^ (μM)	Fold change in sensitivity^*b*^	Amino acid substitution(s) in protease
20	1	14.3 ± 1.7	10.9	A105V/I109V
	2	12.3 ± 1.2	9.4	A105V/I109V
	3	15.3 ± 1.1	11.7	A105V/I109V
15	1	8.5 ± 1.9	6.5	A105V/I109V
	2	14.3 ± 1.3	10.9	A105V/I109V
	3	6.9 ± 1.7	5.3	I109V
10	1	6.6 ± 2.4	5.0	I109V
	2	6.3 ± 2.3	4.8	I109V
	3	5.9 ± 1.3	4.5	I109V
DMSO		1.3 ± 0.2	1.0	

a The EC_50_s are the means ± standard deviations from three independent experiments.

b Fold change in sensitivity relative to that achieved with DMSO.

### Mutations conferring resistance to rupintrivir map to positions near the norovirus protease active site.

**FIG 2:**
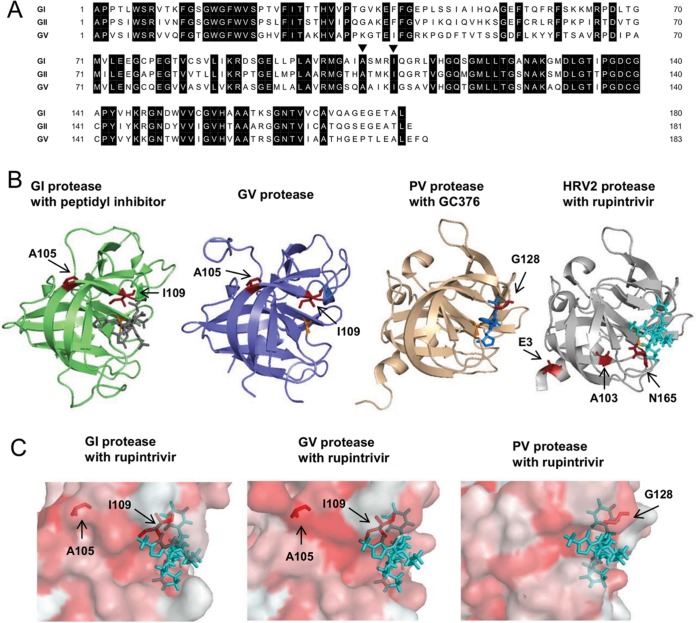
Amino acid sequence alignment and structural comparison of norovirus proteases. (A) Amino acid sequence alignment of the GI (GenBank accession number M87661), GII (GenBank accession number DQ658413), and GV (GenBank accession number DQ285629) proteases used in this study. Identical amino acids (in black) are highlighted. Black arrowheads, positions of the amino acids where substitutions were observed in rupintrivir-resistant replicon cells. (B) Crystal structures of the GI Southampton norovirus protease in complex with a peptidyl inhibitor (gray) (PDB accession number 2IPH) (19) and the GV MNV protease (PDB accession number 4ASH) (20). Residues A105 and I109 in the GI and GV proteases are indicated in red. The crystal structures of poliovirus (PV) protease in complex with GC376 (blue) (PDB accession number 4DCD) (24) and human rhinovirus 2 (HRV2) protease in complex with rupintrivir (cyan) (PBD accession number 1CQQ) (26) are also shown. Residue G128 in the PV protease and residues N165, E3, and A103 in the HRV2 protease, involved in rupintrivir resistance, are indicated in red (23, 25). The catalytic residues C139 (GI), A139 mutated from cysteine (GV), C147 (PV), and C147 (HRV2) are indicated in orange. (C) The crystal structure of the GI, GV, or PV protease was aligned to the HRV2 protease crystal structure in complex with rupintrivir and shown as the hydrophobic surface of each protease with rupintrivir (cyan). Residues A105 and I109 in the GI and GV protease and residue G128 in the PV protease are indicated in red.

An amino acid sequence alignment of HuNoV GI and GII and MNV GV confirmed that A105 and I109 are highly conserved (Fig. 2A). To gain a better understanding of the mechanism by which these mutations might affect rupintrivir binding, we examined the published crystal structures of the GI protease in complex with a peptidyl inhibitor (PDB accession number 2IPH) as well as the GV protease (PDB accession number 4ASH) (19, 20). Analysis of the protease structure revealed that I109 is positioned near the S2 and S4 pockets of the protease (Fig. 2B), regions of the protease that are involved in hydrophobic and van der Waals interactions with the P2 and P4 residues of a substrate or inhibitor (19, 21). A previous mutagenesis study has also suggested that the mutation of I109 affects the binding of the P2 and P4 residues of the substrate (22). The mutations identified here appear to be somewhat similar to those identified in a rupintrivir-resistant poliovirus (PV) strain which had single amino acid substitution, G128Q, in a flexible loop (PDB accession number 4DCD; Fig. 2B) (23, 24) and also in a rupintrivir-resistant HRV2 strain which had the N165T substitution in the S4 pocket (PDB accession number 1CQQ; Fig. 2B) (25, 26). We therefore modeled the interaction of the GI and GV proteases with rupintrivir based on the crystal structure of the HRV2 3C protease with rupintrivir (26) (Fig. 2C). These structural alignments suggested that a change in hydrophobicity associated with the I109V mutation could, in principle, result in the reduced susceptibility of protease to rupintrivir, an effect similar to that caused by the G128Q mutation in rupintrivir-resistant PV protease (Fig. 2C). In contrast, the A105V mutation showed no obvious interactions with rupintrivir in these alignments; however, the possibility of allosteric effects could not be ruled out.

### The mutations I109V and A105V affect the susceptibility to rupintrivir in a cell-based FRET assay.

**FIG 3:**
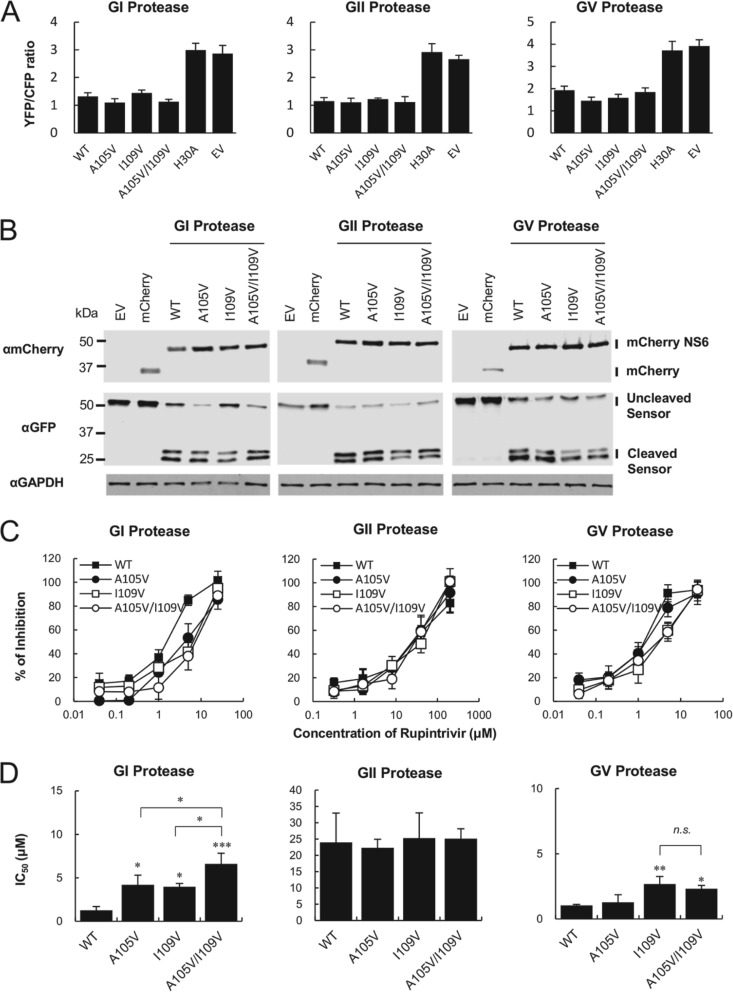
Inhibitory effects of rupintrivir on the mutant proteases in a cell-based FRET assay. (A) The FRET signal from a sensor (YFP/CFP ratio) was lost upon cotransfection with WT or mutant protease but not with the inactive (H30A) protease and the empty vector (EV). The GI, GII, and GV mutant proteases showed cleavage activity comparable to that of the wild type (WT). (B) Western blot analysis of HEK293T cells cotransfected with the GI, GII, or GV NS1/2-NS3 FRET sensor along with either the WT or mutant proteases. Similar cleavage products corresponding to CFP and YFP were observed in the WT and mutant proteases in all genogroups. MM, molecular mass. (C) Dose-response inhibition curves showing the effect of rupintrivir on either the WT or mutant proteases of GI, GII, and GV genotypes in the cell-based FRET assay. (D) IC_50_s of rupintrivir along with the standard deviations of the means from three independent experiments. Asterisks indicate statistically significant differences, as follows: *, *P* < 0.05; **, *P* < 0.01; ***, *P* < 0.001. Abbreviation: n.s., not significant.

Our findings supported the hypothesis that the amino acid positions I109 and A105 could directly or indirectly affect the susceptibility of the viral protease to rupintrivir. To address this hypothesis, we utilized our previously described cell-based FRET assay, which enables the detection of norovirus protease activity in live cells (18). We first sought to examine the impact of the substitutions on the cleavage activity of the viral protease from each genogroup. Therefore, we constructed three mutants, the A105V, I109V, and A105V/I109V mutants, with mutations in each of the GI, GII, and GV proteases. For the FRET substrate, we chose the NS1/2-NS3 cleavage site from the homologous genogroup because our prior study demonstrated that this site was efficiently cleaved by the GI, GII, and GV proteases (18). As expected, a FRET signal was detected in HEK293T cells transfected with the FRET substrate and the empty vector (EV) or an inactive protease which had a H30A substitution in the catalytic site (Fig. 3A). Each mutant protease of all genogroups showed cleavage activity comparable to that of the wild-type (WT) protease. To examine whether the loss of the FRET signal represented cleavage of the substrate, we performed Western blotting analyses on samples transfected with the FRET substrate and protease. Similar cleavage products corresponding to cyan fluorescent protein (CFP) and yellow fluorescent protein (YFP) were observed in all genogroups (Fig. 3B), confirming that the mutations did not have any overt impact on overall protease activity *per se*. Next, we evaluated the inhibitory effects of rupintrivir on WT and mutant proteases in the FRET assay. The GI WT protease was inhibited by rupintrivir in a dose-dependent manner with a 50% inhibitory concentration (IC_50_) value of 1.3 ± 0.4 μM, similar to that seen in the replicon cells (Fig. 3C). In comparison to GI WT protease, a significant reduction in susceptibility to rupintrivir was observed in the GI A105V, I109V, and the A105V/I109V mutant proteases, with IC_50_s of 4.2 ± 1.1 (3.2-fold increase), 4.0 ± 0.4 (3.1-fold increase), and 6.6 ± 1.2 μM (5.1-fold increase), respectively (Fig. 3D). In addition, significant differences were observed when the GI A105V and I109V single mutants were compared with the A105V/I109V double mutant, suggesting that, at least in the context of the GI protease, these substitutions have an additive effect on susceptibility to rupintrivir. In contrast, when the substitutions were introduced into the GII protease, they had no measurable effect on the IC_50_ and only a small, but significant, effect on the GV protease. The GV WT protease showed an IC_50_ of 1.0 ± 0.1 μM, whereas only the mutant proteases with the I109V single substitution and the A105V/I109V double substitution had reduced susceptibility to rupintrivir, with IC_50_s of 2.7 ± 0.6 (2.7-fold increase) and 2.3 ± 0.3 μM (2.3-fold increase), respectively.

### Recombinant MNV containing a single I109V substitution in NS6 showed reduced susceptibility to rupintrivir.

**FIG 4:**
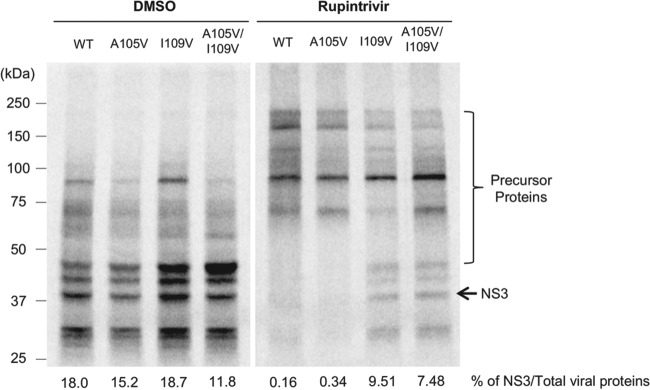
Impact of rupintrivir resistance-conferring mutations on MNV polyprotein processing *in vitro. In vitro* translation reactions were programmed with equal quantities of *in vitro*-transcribed MNV full-length capped RNA containing either the WT or mutant proteases in the presence and absence of 80 μM rupintrivir. The [^35^S]methionine-labeled products were subsequently analyzed by SDS-PAGE and phosphorimaging analysis. The percentage of fully processed NS3, determined on the basis of the expected molecular mass of the proteins, was calculated and expressed relative to the total amount of viral protein produced.

To examine the impact of the rupintrivir resistance mutations in the context of authentic viral replication, we introduced the single mutations A105V and I109V as well as the double mutation A105V/I109V into a full-length infectious MNV-1 cDNA clone (27). To confirm that the introduced mutations had no overt impact on protease activity when expressed in the context of the viral polyprotein, we examined the impact of the mutations on viral polyprotein processing *in vitro*. To this end, we generated *in vitro*-transcribed, capped RNA transcripts from the WT MNV-1 cDNA clone or clones carrying the rupintrivir resistance mutations and examined their translation profiles in rabbit reticulocyte lysates. We found that the processing of the mutants was largely indistinguishable from that of the WT virus (Fig. 4). Furthermore, we demonstrated that the resistance to rupintrivir was maintained when the protease was expressed from the polyprotein as the I109V single mutant, and the A105V/I109V double mutant was still able to produce mature NS3 at a concentration that prevented the cleavage of the polyprotein by the WT protease (Fig. 4).

**FIG 5:**
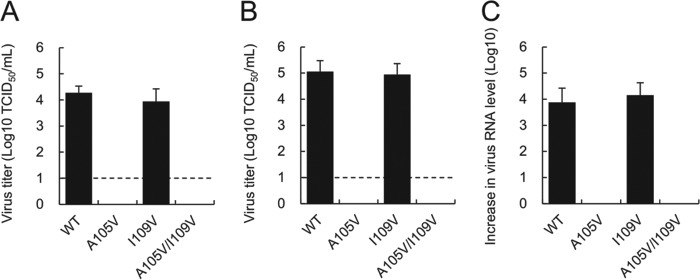
Impact of rupintrivir resistance-conferring mutations on the recovery of recombinant MNV. (A and B) Virus titers obtained at 24 h after transfection of BSR-T7 cells with MNV cDNA (A) or *in vitro*-transcribed and capped MNV RNA (B). The virus titers in BV-2 cells were determined by the TCID_50_ method. Error bars represent means ± standard deviations from three independent experiments. Dotted lines, detection limit of the assay. (C) The increase in the RNA level (the RNA level at 12 h postinoculation minus the RNA level at 1 h postinoculation) was determined by qRT-PCR after three passages with BV-2 cells. The supernatant of BSR-T7 cells transfected with MNV cDNA was inoculated onto BV-2 cells. At 48 h postinoculation, the supernatant of BV-2 cells was next inoculated onto BV-2 cells. This passage was repeated two times. After three passages, the supernatant was inoculated onto BV-2 cells and the increase in the RNA level was measured. No increase in the amount of viral RNA of the A105V and A105V/I109V recombinant viruses was observed.

To examine the impact of the mutations on viral resistance to rupintrivir, we used the cDNA clones to generate recombinant viruses following transfection of cells expressing T7 RNA polymerase. The WT and I109V recombinant viruses were successfully recovered in BSR-T7 cells, while the A105V and A105V/I109V recombinant viruses were nonviable (Fig. 5A). As we have previously demonstrated that RNA-mediated reverse genetics can improve the yield of recovered viruses, particularly those with reduced fitness (28), we also examined the ability of *in vitro*-transcribed and capped RNA to generate recombinant virus. As expected, the virus yield observed for the WT and I109V recombinant viruses was significantly higher than that obtained using a DNA-based reverse genetics system, while the A105V and A105V/I109V viruses remained nonviable (Fig. 5B). Since there is a possibility that the recombinant viruses with A105V replicate without inducing a cytopathic effect, which is used as the basis of the infectivity titration assay, we measured the levels of viral RNA in samples by qRT-PCR after three passages in culture. Despite repeated passage, we found no signs of viral RNA replication from the A105V and A105V/I109V recombinant viruses (Fig. 5C). These results suggest that amino acid residue A105 plays a critical role in virus replication in the MNV strain used in this study.

**FIG 6:**
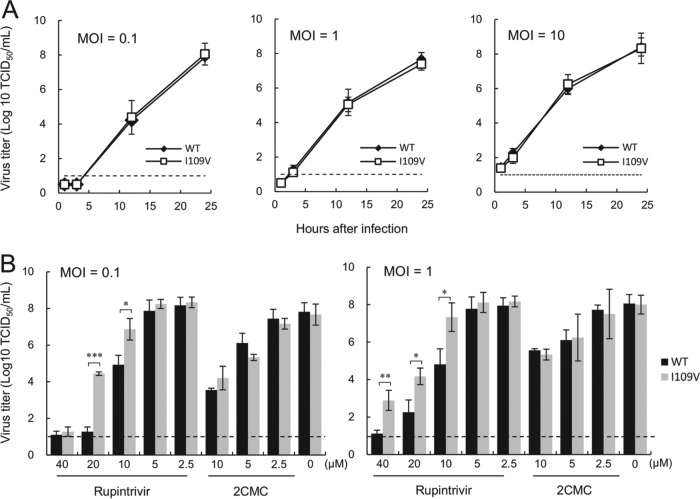
The I109V substitution in the MNV protease results in reduced susceptibility to rupintrivir during viral replication in cell culture. (A) The growth kinetics of the WT and I109V recombinant MNV were assessed following infection of BV-2 cells at MOIs of 0.1, 1, and 10 TCID_50_/cell. Viral infectivity levels were determined by measuring the release of infectious virus into the culture supernatant at various times postinfection by determination of the TCID_50_. (B) The inhibitory effects of rupintrivir and 2CMC against either the WT or the recombinant MNV I109V mutant were assessed following infection of BV-2 cells at MOIs of 0.1 and 1 TCID_50_/cell. A significant difference in viral titers following treatment with 10 and 20 μM and 10, 20, and 40 μM rupintrivir was observed between the WT and I109V recombinant viruses at MOIs of 0.1 and 1, respectively (*, *P* < 0.05; **, *P* < 0.01; ***, *P* < 0.001). In contrast, there were no significant differences in virus titers following treatment with 2CMC.

The impact of the introduced mutations on viral replication in the absence of rupintrivir was analyzed by comparing the viral growth kinetics of the WT and I109V recombinant viruses at various multiplicities of infection (MOIs) of 0.1, 1, and 10 50% tissue culture infective doses (TCID_50_)/cell (Fig. 6A). The replication of the I109V recombinant virus was indistinguishable from that of the WT virus at all the MOIs studied. Furthermore, sequence analysis indicated that the I109V recombinant virus was genetically stable, even after multiple passages in cell culture. We then evaluated the inhibitory effects of rupintrivir and, as a control, the nucleoside viral polymerase inhibitor 2-*C*-methylcytidine (2CMC), against the WT and I109V recombinant viruses. Significant differences in viral titers following treatment with 10 and 20 μM rupintrivir were observed between the WT and I109V recombinant viruses at two MOIs (Fig. 6B). In addition, a small, but significant, reduction in the susceptibility of the I109V virus to rupintrivir was shown at MOIs of 0.1 and 1 TCID_50_/cell, with the EC_90_s increasing from 9.1 to 18.1 μM (2.0-fold increase; *P* < 0.001) and 8.9 to 17.0 μM (1.9-fold increase; *P* < 0.001), respectively. There was no significant difference in the EC_90_ values for 2CMC between the I109V and WT viruses. These results demonstrate that the I109V substitution alone is sufficient to impact susceptibility to rupintrivir in the context of the MNV protease.

## DISCUSSION

Currently, there are no antiviral drugs for HuNoV, and as such, antiviral-resistant mutants of HuNoV have yet to pose a major clinical problem. However, a better understanding of the mechanisms of resistance to various classes of antiviral drugs is critical for developing ways of minimizing the appearance of resistance. The lack of a robust culture system for HuNoV has severely limited the development of therapeutic approaches, but with the recent discovery of the B cell and enteroid culture systems, this bottleneck now appears to have been overcome. Despite this, to date, most studies have relied on the use of a replicon-based approach due to the scalability and the robust nature of the assay. However, the mechanism by which drug resistance may occur in HuNoV has yet to be described. In the present study, we successfully generated a replicon system in a physiologically relevant cell line, namely, human gastric tumor cells (29). This was then used to isolate replicon cells with reduced susceptibility to rupintrivir. The EC_50_ and CC_50_ values of rupintrivir in the HGT-NV replicon system were 1.3 ± 0.1 μM and >100 μM, respectively, which were comparable to those presented in a previous report describing a study with an HG23 cell system based on the same replicon in the Huh-7 hepatocellular carcinoma cell line (24). The inhibitory effects and the high selectivity index (CC_50_/EC_50_) of >77 were considered sufficient to perform the resistance study. Repeated cultivation of the replicon cells for 45 days in the presence of rupintrivir resulted in the selection of two amino acid substitutions, I109V and A105V/I109V, in GI protease. The precise time during the culture period when a mutation first arose was not identified in this study, as the goal was to simply identify mutations that conferred high levels of resistance to rupintrivir. The mutant with the double amino acid A105V/I109V substitution showed a tendency to be less susceptible to rupintrivir than the mutant with the I109V single amino acid substitution (Table 1). Similar features of resistance have been reported in an *in vitro* study with rupintrivir-resistant HRV2 (25). Serial passage of HRV2 for 62 days resulted in the single amino acid substitution N165T and double amino acid substitutions (N165T/E3G or N165T/A103V), and the mutants with these substitutions showed 2- and 5-fold reduced susceptibility to rupintrivir, respectively. In general, the resistance to protease inhibitors typically involves the accumulation of primary and secondary mutations that are located within and outside the active site of protease (30, 31). Although the process of accumulation of mutations was not clarified in this study, on the basis of the observation that the A105V substitution was not identified in isolation, it is presumed that it arose as a secondary mutation that can cause increased levels of resistance and/or compensate for some reduced functionality of the protease.

Amino acid sequence alignment revealed that A105 and I109 are absolutely conserved in the GI, GII, and GV genogroups (Fig. 2A). These amino acid positions were also conserved in the HuNoV GIV genogroup, which is rarely detected, whereas the bovine norovirus GIII and canine norovirus GVI genogroups had T105 and V109 in the viral protease and A105 and V109 in the viral protease, respectively (data not shown). Using a cell-based FRET assay with mutant proteases from the GI, GII, or GV genogroup, we demonstrated that the mutant proteases had proteolytic activity on an NS1/2-3 cleavage site-based substrate equivalent to that of the WT protease (Fig. 3A and B). We elucidated that the GI I109V and A105V/I109V substitutions found in replicon cells contributed directly to the reduced susceptibility to rupintrivir in the FRET assay (Fig. 3C and D). Notably, we found that the GI A105V mutant protease also showed reduced susceptibility to rupintrivir, suggesting that, at least in the context of the GI protease, the A105V substitution may affect the interaction with rupintrivir. In contrast, the A105V substitution in the GV protease had no appreciable impact on susceptibility to rupintrivir, which was consistent with results obtained using *in vitro* translation (Fig. 3D and 4). These results indicate that the A105 residue in GV protease is unlikely to be involved in the interaction with rupintrivir. This hypothesis was supported by the IC_50_s of the GV A105V/I109V double mutant, which were comparable to those of the GV I109V single mutant (Fig. 3D). Surprisingly, no significant differences in the inhibitory effects of rupintrivir were observed between the WT GII protease and proteases carrying any of the mutations. In addition, the susceptibility of the GII protease to rupintrivir was substantially lower than that of the GI and GV WT proteases. These findings suggest that the GII protease has a weaker affinity for rupintrivir than the GI and GV proteases, probably because the binding site is less conserved and the effects of susceptibility to rupintrivir by amino acid substitution are different among the different genotypes.

We obtained similar changes in sensitivity to rupintrivir when the mutations were introduced into the recombinant MNV clones, where the MNV clone with the I109V mutation showed reduced susceptibility to rupintrivir in cell culture but viral fitness was not overtly affected. Surprisingly, while the I109V mutant was readily recovered using two different reverse genetics approaches, mutants with the A105V and A105V/I109V substitutions were not viable (Fig. 6). Our FRET assays and *in vitro* translations assays confirmed that the mutants with the single substitution A105V and the double substitution A105V/I109V had no defect in protease activity *per se* (Fig. 3A and 4), suggesting that the inability to recover viable virus may be related to some other function of the protease. While the other functions that the HuNoV protease plays in the viral life cycle have yet to be fully elucidated, previous work has demonstrated that the HuNoV protease has RNA binding activity (32), as well as the ability to cleave cellular protease substrates (33). Work on the closely related poliovirus protease has also identified a phosphoinositide binding domain (34), but it remains to be determined whether the A105V mutation affects either of these or other as yet to be identified functions. It is noteworthy that the A105V mutation was not detected in isolation and was found only in combination with I109V. While it was outside the scope of the current study, it may be possible to use the MNV infectious culture system to generate secondary mutations in other NS proteins that could compensate for the A105V change introduced in the protease.

Although the crystal structures of proteases from different strains of norovirus are available (19, 21, 35, 36), in the absence of any crystal structure of a norovirus protease in complex with rupintrivir, the impact of these amino acid substitutions remains unclear. The crystal structure of the Norwalk virus protease revealed that the I109 residue is positioned in the S2 pocket, which is formed by residues I109, Q110, R112, and V114, and also the S4 pocket, which is formed by residues M107, I109, T161, T166, and V168 and which is considered to be involved in hydrophobic interactions with a substrate or rupintrivir (21, 36). In addition, the essential role of I109 of the S2 pocket in the binding of both P2 and P4 residues has previously been reported (22). The S2 pocket of the protease is also known to undergo conformational changes in response to the binding substrates (21). We therefore modeled the complex of the GI protease with rupintrivir based on the crystal structure of the HRV2 proteases (Fig. 2C) (10, 21). The structural alignments suggest that the GI and GII I109V substitution may affect the hydrophobic interactions of protease with rupintrivir. The mechanism of rupintrivir resistance generated in poliovirus appears to be somewhat similar, as the G128Q substitution in a flexible loop of the poliovirus 3C protease has a similar impact on susceptibility to rupintrivir (Fig. 2B) (23). Like the E3G and A103V substitutions in the 3C protease of rupintrivir-resistant HRV2, the A105V substitution was not located close to a location of the interaction of protease with rupintrivir (Fig. 2B) (25). These results may imply that these substitutions are not the primary mutations conferring resistance but are in fact secondary mutations. Moreover, despite the accumulation of multiple amino acid substitutions, high-level resistance to rupintrivir did not occur. This highlights the suggestion that there is a significant barrier to rupintrivir resistance, further supporting the possibility that viral protease is a potential therapeutic antiviral target for noroviruses.

In conclusion, we successfully identified, for the first time, a protease inhibitor-resistant HuNoV replicon, indicating at least one potential route to rupintrivir resistance. Furthermore, we demonstrated that rupintrivir has a relatively high genetic barrier, adding further support to the use of protease inhibitors as potential therapeutics against noroviruses.

## MATERIALS AND METHODS

### Cells.

A human gastric tumor-1 (HGT-1) cell line (29) was used to establish a human norovirus replicon-harboring HGT (HGT-NV) cell line. In brief, viral protein genome (VPg)-linked RNA was isolated from BHK cells containing the Norwalk virus GI replicon and transfected into HGT-1 cells. HGT-NV replicon cells were selected in G418 (1 mg/ml), and limiting dilution was used to identify a clone with high viral RNA levels. The generated HGT-NV replicon cells were grown and maintained under G418 selection. The replicon-containing BHK cell line was generated by the transfection of *in vitro*-transcribed capped RNA from the cDNA construct of the Norwalk virus replicon (9), kindly provided by Kim Green (National Institutes of Health, Bethesda, MD, USA), into baby hamster kidney cells. Human embryonic kidney HEK293T cells were obtained from ATCC. The murine microglial BV-2 cell line was provided by Jennifer Pocock (University College London, London, UK). BSR-T7 cells (baby hamster kidney cells engineered to express T7 polymerase) were provided by Karl-Klaus Conzelmann (Ludwig Maximilian University, Munich, Germany). Cells were maintained in complete Dulbecco's minimal essential medium (DMEM) containing 10% fetal bovine serum, penicillin (100 units/ml), streptomycin (100 μg/ml), and, in the case of HGT-NV and BSR-T7 cells, G418 (0.5 mg/ml).

### Compounds.

Rupintrivir and 2-*C*-methylcytidine (2CMC) were purchased from Sigma-Aldrich and dissolved in dimethyl sulfoxide (DMSO). A final DMSO concentration of 1% was used in all cell culture experiments in this study.

### Plasmids.

The generation of plasmids expressing norovirus protease from GI.1 (M87661), GII.4 (DQ658413), or GV (DQ285629) norovirus and FRET assay constructs has been described previously (18). The plasmids carrying GI, GII, and GV proteases with the A105V, I109V, or A105V/I109V mutation were generated from the wild-type (WT) protease constructs described above by the QuikChange site-directed mutagenesis method. Mutant MNV genomes for reverse genetics were generated by two-step PCR mutagenesis of the WT MNV genome (pT7 MNV 3RZ) construct as described previously (27). The primers used for generation of the plasmids are listed in Tables S1 and S2 in the supplemental material.

### Inhibitory effect of rupintrivir on viral replication in replicon cells.

The inhibitory effect of rupintrivir on HuNoV replication was assessed by quantification of the levels of GI NuNoV RNA and β-actin RNA as the reference gene. Briefly, HGT-NV cells were plated into 96-well plates at a density of 5 × 10^3^ cells/well in complete DMEM without G418 and then incubated for 24 h in a CO_2_ incubator at 37°C. After incubation, serial dilutions of rupintrivir were added to the cells, and then the cells were incubated for 72 h. After incubation, the supernatant was removed and the cells were washed with phosphate-buffered saline (PBS), and then total RNA was extracted using a GenElute system (Sigma-Aldrich). The real-time quantitative reverse transcription-PCR (qRT-PCR) assay was performed by using a One-Step Plus qRT-PCR kit according to the protocol of the manufacturer (Primer Design). Cycling conditions were reverse transcription at 55°C for 30 min and initial denaturation at 95°C for 5 min, followed by 40 cycles of denaturation at 95°C for 15 s and annealing and extension at 60°C for 1 min (ViiA 7 real-time PCR system; Applied Biosystems, USA). The sequences of the primers and probes that were used for qRT-PCR are as follows (10, 37): for GI HuNoV RNA, 5′-CGY TGG ATG CGN TTY CAT GA-3′ for the forward primer, 5′-CTT AGA CGC CAT CAT CAT TYA C-3′ for the reverse primer, and 5′-FAM-AGA TCG CGG TCT CCT GTC CA-TAMRA-3′ (where FAM is 6-carboxyfluorescein and TAMRA is 6-carboxytetramethylrhodamine) for the probe, and for human β-actin RNA, 5′-GGC ATC CAC GAA ACT ACC TT-3′ for the forward primer, 5′-AGC ACT GTG TTG GCG TAC AG-3′ for the reverse primer, and 5′-HEX-ATC ATG AAG TGT GAC GTG GAC ATC CG-TAMRA-3′ (where HEX is 6-carboxy-2,4,4,5,7,7-hexachlorofluorescein) for the probe. Relative quantification was performed using the 2^−ΔΔ*CT*^ threshold cycle (*C_T_*) method (38). The 50% effective concentration (EC_50_) values of rupintrivir were calculated using XLfit (version 5.3) software.

### Cell cytotoxicity of rupintrivir in replicon cells.

The cell cytotoxicity of rupintrivir against HGT-NV cells was determined by a resazurin assay (39). Briefly, subconfluent HGT-NV cells in 96-well plates were treated with various concentrations of rupintrivir and then incubated for 72 h in a CO_2_ incubator at 37°C. After incubation, the supernatant was removed and the cells were washed with PBS, and then complete DMEM containing resazurin (0.1 mg/ml) was added to the cells. After 4 h of incubation, the optical density at 560 nm with a 620-nm optical density reference was determined by using a SpectraMax i3 instrument (Molecular Devices). The 50% cytotoxic concentration (CC_50_) values of rupintrivir were calculated using XLfit (version 5.3) software.

### Colony formation assays.

HGT-NV cells treated with rupintrivir or DMSO for 12 days in the absence of G418 were plated into 6-well plates in complete DMEM and maintained without rupintrivir in the presence of G418 (1.5 mg/ml). After 1 week of incubation, the colonies on the plate were stained with 0.1% toluidine blue and photographed.

### Sequence analysis.

Total RNA was purified from replicon cells by using a GenElute system (Sigma-Aldrich), and then cDNA was synthesized from the RNA by using Moloney murine leukemia virus reverse transcriptase (Invitrogen) and a random hexamer. The protease-coding region was amplified by PCR with *Taq* DNA polymerase (Invitrogen) and the specific primers listed in Table S3. The amplified DNA was sequenced in an Applied Biosystems 3730xl DNA analyzer. The sequences of the protease-coding region from rupintrivir-resistant replicon cells were compared with those from the replicon cells treated with DMSO, and amino acid substitutions were analyzed.

### Cell-based FRET assay.

The YFP/CFP ratio was used to determine FRET efficiency, as described previously (18, 40). Briefly, HEK293T cells were plated into black 96-well cell culture microplates (Greiner Bio-One) at a density of 5 × 10^4^ cells/well in 100 μl of DMEM containing 10% fetal bovine serum with or without various concentrations of rupintrivir and then incubated for 4 h in a CO_2_ incubator at 37°C. After incubation, the cells were transfected with 0.1 μg of protease and 0.1 μg of the appropriate FRET construct by transfection with the Lipofectamine 2000 reagent according to the manufacturer's protocol (Invitrogen) and then incubated for 24 h. After incubation, the medium was removed and replaced with PBS. The plates were then analyzed on a SpectraMax i3 instrument in fluorescence mode, with excitation set at 434 nm and the reading emissions set at 477 nm (CFP) and 527 nm (YFP). The background fluorescence from wells transfected with pcDNA3.1 was determined and subtracted. The 50% inhibitory concentration (IC_50_) values of rupintrivir were calculated using XLfit (version 5.3) software.

### Reverse genetics of MNV.

Reverse genetics rescues of WT or mutant MNV (with the A105V, I109V, and A105V/I109V mutations in protease NS6) were performed by two methods as described previously (27, 41). Briefly, BSR-T7 cells were infected with recombinant fowlpox virus expressing T7 polymerase. At 3 h postinfection, the cells were transfected with plasmids containing the MNV genome, either the WT MNV genome or the genomes of the various mutant MNV constructs, under the control of a T7 promoter. In the case of the RNA-based MNV reverse genetics system, BSR-T7 cells seeded 24 h prior to transfection were typically transfected with capped *in vitro*-synthesized MNV RNA. At 24 h posttransfection, the cells were frozen at −80°C for viral titer determination or processed for repetitive passages. The viral titer was determined from the 50% tissue culture infective dose (TCID_50_) in BV-2 cells. Repetitive passage was also performed with the supernatant of BSR-T7 cells transfected with the MNV plasmid in BV-2 cells. At 48 h postinoculation, the supernatant of BV-2 cells was inoculated onto BV-2 cells. Virus was passaged in BV-2 cells a further two times. After three passages, the supernatants were inoculated onto BV-2 cells and the increase in RNA level (the RNA level at 12 h postinoculation minus the RNA level at 1 h postinoculation) was determined by qRT-PCR. The sequences of the primers and probes that were used for qRT-PCR of MNV RNA are as follows (42): 5′-CCG CAG GAA CGC TCA GCA G-3′ for the forward primer, 5′-GGY TGA ATG GGG ACG GCC-3′ for the reverse primer, and 5′-FAM-ATG AGT GAT GGC GCA-TAMRA-3′ for the probe.

### Antiviral effects of compounds against MNV infection.

BV-2 cells were infected with recombinant WT MNV or the MNV I109V mutant at an MOI of 0.1 or 1 for 1 h in the CO_2_ incubator at 37°C. After incubation, the supernatant was removed and the cells were washed twice with PBS, and then complete DMEM containing various concentrations of rupintrivir or 2CMC was added to the cells. After 24 h for incubation, the virus titers of the supernatant were determined by measurement of the TCID_50_. On the basis of the TCID_50_, the 90% effective concentration (EC_90_) values of rupintrivir and 2CMC were calculated using XLfit (version 5.3) software.

### Western blot analysis.

Anti-GAPDH (glyceraldehyde-3-phosphate dehydrogenase; catalog number AM4300), anti-green fluorescent protein (GFP; catalog number ab290), and anti-mCherry (catalog number LS-C204825) antibodies were purchased from Ambion, Abcam, and LifeSpan BioSciences, respectively. IRdye 800 secondary antibodies were purchased from LI-COR. HEK293T cells cotransfected with the GI, GII, or GV NS1/2-NS3 FRET sensor and protease were lysed in radioimmunoprecipitation assay buffer (50 mM Tris-HCl [pH 8.0], 150 mM NaCl, 1 mM EDTA, 1% Triton X-100, 0.1% SDS). Protein concentrations were determined by use of a Pierce bicinchoninic acid protein assay kit. Samples were separated by SDS-PAGE using 12.5% polyacrylamide gels and transferred to 0.2-μm-pore-size nitrocellulose membranes. Blocking and staining steps were carried out in 5% nonfat dried milk in PBS containing 0.2% Tween 20. Membranes were imaged through detection of far-red fluorescence on a LI-COR Odyssey imager.

### *In vitro* translation assay.

*In vitro* translation reactions were performed using a Flexi rabbit reticulocyte lysate system (Promega). The reaction mixtures were prepared according to the manufacturer's instructions as described for capped mRNAs and programmed with 1 μg of RNA per 25-μl reaction mixture. The reaction mixtures were incubated at 30°C for 90 min prior to the addition of an equal volume of SDS-PAGE sample buffer. Samples were subsequently resolved on a 12.5% SDS-polyacrylamide gel before exposure to a phosphorimager screen.

### Structural comparison and alignments of protease.

The crystal structure of the protease of HuNoV GI (PDB accession number 2IPH) (19), MNV GV (PDB accession number 4ASH) (20), or poliovirus (PV) (PDB accession number 4DCD) (24) was aligned to the human rhinovirus 2 (HRV2) protease crystal structure (PDB accession number 1CQQ) (26). Residues A105 and I109 in the GI and GV proteases, residues N165, E3, and A103 in the HRV2 protease, and residue G128Q in the PV protease involved in rupintrivir resistance are shown as sticks and in red in Fig. 2. The catalytic residues C139 (GI), A139 mutated from cysteine (GV), C147 (PV), and C147 (HRV2) are indicated in orange in Fig. 2. The crystal structure of the GI, GV, or PV protease was aligned to the HRV2 protease crystal structure in complex with rupintrivir and is shown as the hydrophobic surface of each protease with rupintrivir (cyan) in Fig. 2. All structure alignments and comparisons were performed using the PyMOL molecular graphics program.

### Statistical analysis.

Statistical comparisons employed parametric evaluation using one-way analysis of variance or Student's *t* test in GraphPad Prism (version 6) software. *P* values below 0.05 were considered statistically significant.

## Supplementary Material

Supplemental material
