# Supplementary material for: Selection and Characterization of Rupintrivir-Resistant Norwalk Virus Replicon Cells In Vitro
Source: Antimicrob Agents Chemother. 2018 Apr 26;62(5):e00201-18. doi: 10.1128/AAC.00201-18 (PMC5923142; doi:10.1128/AAC.00201-18)
Supplement: Supplemental material [file supp_62_5_e00201-18__index.html]

Supplemental material 

# Selection and Characterization of Rupintrivir-Resistant Norwalk Virus Replicon Cells *In Vitro*

## Supplemental material

- Supplemental file 1 -

  Supplemental Tables S1 to S3

  PDF, 81K
