# Supplementary material for: Selection and Characterization of Rupintrivir-Resistant Norwalk Virus Replicon Cells In Vitro
Source: Antimicrob Agents Chemother. 2018 Apr 26;62(5):e00201-18. doi: 10.1128/AAC.00201-18 (PMC5923142; doi:10.1128/AAC.00201-18)
Supplement: Supplemental material [file AAC.00201-18_zac005187162s1.pdf]

Table S1. Sequence of primers used for generation of FRET assay constructs in QuikChange site-directed mutagenesis method

Genogroup	Mutation site in NS6	Forward primer (5'-3')	Reverse primer (5'-3')
GI	A105V	TATGGGGGCTATTGTCTCCATGAGGATAC	GTATCCTCATGGAGACAATAGCCCCCATA
	I109V	TTGCCTCCATGAGGGTACAGGGTCGGCTT	AAGCCGACCCTGTACCCTCATGGAGGCAA
	A105V/I109V	GGGCTATTGTCTCCATGAGGGTACAGGGTCGGC	GCCGACCCTGTACCCTCATGGAGACAATAGCCC
GII	A105V	AATGGGGACCCATGTAACCATGAAAATCC	GGATTTTCATGGTTACATGGGTCCCCATT
	I109V	ATGCAACCATGAAAGTCCAAGGGCGCACT	AGTGCGCCCTTGGACTTTCATGGTTGCAT
	A105V/I109V	GGACCCATGTAACCATGAAAGTCCAAGGGCGCA	TGCGCCCTTGGACTTTCATGGTTACATGGGTCC
GV	A105V	GATGGGTTCACAGGTCGCCATCAAGATTG	CAATCTTGATGGCGACCTGTGAACCCATC
	I109V	AGGCCGCCATCAAGGTTGGTAGTGCCGTT	AACGGCACTACCAACCTTGATGGCGGCCT
	A105V/I109V	GTTCACAGGTCGCCATCAAGGTTGGTAGTGCCG	CGGCACTACCAACCTTGATGGCGACCTGTGAAC

Table S2. Sequence of primers used for generation of mutant MNV plasmid in two-step PCR mutagenesis method

	Mutation site in NS6	Name	Forward primer (5'-3')	Name	Reverse primer (5'-3')
1st PCR	A105V	MNV/2392F	GTGTCAGAAGGATAAAGGAGGCCCGCCTCCGCTGC	A105V_R	CAATCTTGATGGCGACCTGTGAACCCATC
		A105V_F	GATGGGTTCACAGGTCGCCATCAAGATTG	MNV/3734R	CGCGTGGTTCTGAATAGGGCTTAAGCTGATCTCGC
	I109V	MNV/2392F	GTGTCAGAAGGATAAAGGAGGCCCGCCTCCGCTGC	I109V_R	AACGGCACTACCAACCTTGATGGCGGCCT
		I109V_F	AGGCCGCCATCAAGGTTGGTAGTGCCGTT	MNV/3734R	CGCGTGGTTCTGAATAGGGCTTAAGCTGATCTCGC
	A105V/I109V	MNV/2392F	GTGTCAGAAGGATAAAGGAGGCCCGCCTCCGCTGC	A105V/I109V_R	CGGCACTACCAACCTTGATGGCGACCTGTGAAC
		A105V/I109V_F	GTTCACAGGTCGCCATCAAGGTTGGTAGTGCCG	MNV/3734R	CGCGTGGTTCTGAATAGGGCTTAAGCTGATCTCGC
2nd PCR	A105V<br>I109V<br>A105V/I109V	MNV/2392F	GTGTCAGAAGGATAAAGGAGGCCCGCCTCCGCTGC	MNV/3734R	CGCGTGGTTCTGAATAGGGCTTAAGCTGATCTCGC

Table S3. Sequence of primers used for PCR and sequencing

Name	Forward primer (5'-3')	Name	Reverse primer (5'-3')
NV/1001F	TTGGTGTCGCGGCCATTTAAAGATC	NV/3500R	TCAAGGACCATACCTGTCAAGTCAG
NV/2001F	TTGTCAACCTCGGGCCTGTTTGCCG	NV/4001R	GTTGTAGGGATGGGCCATTCTGTAC
NV/2501F	GCCAGTGTACAAATCAAAGAAGACA		
NV/3501F	CTGACTTGACAGGTATGGTCCTTGA		
